# Impact of chronic biomass smoke exposure on pulmonary function and respiratory health in elderly women: A rural perspective

**DOI:** 10.5578/tt.2025011018

**Published:** 2025-03-24

**Authors:** Burcu TURAN, Yeliz ÇELİK, Şehnaz OLGUN YILDIZELİ, Yağmur KAPTAN, Baran BALCAN

**Affiliations:** 1 Clinic of Pulmonology, Bilecik Teaching and Education Hospital, Bilecik, Türkiye; 2 Department of Neuroscience, Koç University Research Center for Translational Medicine, İstanbul, Türkiye; 3 Department of Pulmonology and Intensive Care, Marmara University Faculty of Medicine, İstanbul, Türkiye; 4 Clinic of Pulmonology, Bayraklı City Hospital, İzmir, Türkiye; 5 Department of Pulmonology, Koç University Faculty of Medicine, İstanbul, Türkiye

## Abstract

**ABSTRACT**

**
Impact of chronic biomass smoke exposure on pulmonary function and respiratory health in elderly women: A rural perspective
**

**Introduction:** *Chronic exposure to biomass smoke is a significant health concern, particularly in rural areas where women are primarily responsible for household cooking using organic fuels. This study aimed to evaluate the impact of biomass smoke exposure on pulmonary function and its associated risk factors among elderly women.
*

**Materials and Methods:** *A total of 474 women were screened, of whom 115 were exclusively exposed to biomass smoke, and 32 participants aged ≥65 years were included in the elderly cohort. Pulmonary function tests were performed using a calibrated spirometer, assessing reduced forced expiratory volume in 1 second (FEV1), forced vital capacity (FVC), and FEV1/FVC ratio. Logistic and linear regression analyses were conducted to explore the relationship between pulmonary outcomes and exposure duration, demographic characteristics, and comorbidities.
*

**Results:** *Among all participants, obstructive lung disease (FEV1/FVC < 70%) was observed in 84.3%, while restrictive patterns were found in 16.7%. Prolonged biomass smoke exposure was significantly associated with lower FEV1, FVC, and FEV1/FVC ratios (p< 0.001). Multivariate logistic regression identified exposure duration as an independent predictor of obstruction [Odds ratio (OR)= 1.25, 95% confidence interval (CI)= 1.08-1.44, p< 0.001]. In the elderly subgroup, logistic regression confirmed a significant association between biomass exposure duration and obstruction (OR= 1.26, 95% CI= 1.02-1.57, p= 0.035), while linear regression revealed a negative correlation between exposure duration and FEV1/FVC ratio (β= -0.65, 95% CI= -0.09 to -0.03, p< 0.001).
*

**Conclusion:** *Prolonged exposure to biomass smoke is strongly associated with deteriorated pulmonary function, particularly among elderly women. These findings underscore the urgent need for targeted interventions to reduce biomass smoke exposure and improve respiratory health in vulnerable rural populations.
*

**Key words:** *Biomass; pulmonary function test; chronic obstructive pulmonary disease
*

**ÖZ**

**
Kronik biyokütle dumanına maruz kalan yaşlı kadınlarda akciğer fonksiyonu ve solunum sağlığı üzerindeki etkisi: Kırsal perspektif
**

**Giriş:** *Biyokütle dumanına kronik maruziyet, özellikle kırsal bölgelerde organik yakıtlarla yemek pişiren kadınlar için önemli bir sağlık sorunudur. Bu çalışma, biyokütle dumanına maruziyetin yaşlı kadınlarda akciğer fonksiyonları üzerindeki etkisini ve ilişkili risk faktörlerini değerlendirmeyi amaçlamaktadır.
*

**Materyal ve Metod:** *Toplam 474 kadın taranmış, bunlardan yalnızca biyokütle dumanına maruz kalan 115 kişi seçilmiştir. Çalışmaya, 65 yaş ve üzeri 32 katılımcı yaşlılar grubuna dahil edilmiştir. Akciğer fonksiyon testleri, zorlu ekspirasyonun birinci saniyedeki volümü (FEV1), zorlu vital kapasite (FVC) ve FEV1/FVC oranını değerlendiren kalibre edilmiş bir spirometre kullanılarak gerçekleştirilmiştir. Akciğer fonksiyonları ile maruziyet süresi, demografik özellikler ve komorbiditeler arasındaki ilişkiyi incelemek için lojistik ve doğrusal regresyon analizleri yapılmıştır.
*

**Bulgular:** *Obstrüktif akciğer hastalığı (FEV1/FVC< %70), tüm katılımcılar arasında %84.3 oranında gözlenirken, restriktif paternler %16.7 oranında tespit edilmiştir. Uzun süreli biyokütle dumanı maruziyeti, FEV1, FVC ve FEV1/FVC oranlarının düşüklüğü ile anlamlı şekilde ilişkili bulunmuştur (p< 0.001). Çok değişkenli lojistik regresyon analizine göre, maruziyet süresi obstrüksiyonun bağımsız bir belirleyicisi olarak tanımlanmıştır [Olabilirlik oranı testi (OOT)= 1,25; %95 güven aralığı (GA)= 1.08-1.44; p< 0.001). Yaşlı alt grupta yapılan lojistik regresyon analizi, biyokütle maruziyet süresi ile obstrüksiyon arasında anlamlı bir ilişki göstermiştir (OOT= 1.26; %95 GA= 1.02-1.57; p= 0,035). Doğrusal regresyon analizi ise maruziyet süresi ile FEV1/FVC oranı arasında negatif bir korelasyon ortaya koymuştur (β= -0.65; %95 GA= -0.09 ile -0.03; p< 0.001).
*

**Sonuç:** *Uzun süreli biyokütle dumanı maruziyeti, özellikle yaşlı kadınlarda akciğer fonksiyonlarının bozulmasıyla güçlü bir şekilde ilişkilidir. Bu bulgular, biyokütle dumanına maruziyeti azaltmaya yönelik hedefli müdahalelerin gerekliliğini ve kırsal bölgelerde yaşayan kırılgan nüfusun solunum sağlığını iyileştirmek için acil önlemler alınması gerektiğini vurgulamaktadır.
*

**Anahtar kelimeler:** *Biyokütle; solunum fonksiyon testi; kronik obstrüktif akciğer hastalığı
*

## INTRODUCTION

Chronic obstructive pulmonary disease (COPD) is a significant global health concern, particularly in low- and middle-income countries where biomass fuel usage is prevalent (1,2). In these regions, women are disproportionately affected due to their primary role in cooking with solid fuels such as wood, charcoal, and dung in poorly ventilated environments (3). This exposure leads to substantial respiratory health chal- lenges, including the development of COPD, which is among the leading causes of death worldwide (4). Biomass smoke contains harmful substances such as particulate matter (PM), carbon monoxide (CO), nitrogen oxides, and volatile organic compounds (5). Prolonged exposure to these pollutants has been associated with both obstructive and restrictive lung diseases, mirroring the pathophysiology observed in cigarette smoke-induced lung damage (3,6). The mechanisms by which biomass smoke leads to res- piratory disease include chronic inflammation and oxidative stress in the lungs, resulting in obstruction and restriction of airflow (7). Furthermore, biomass smoke exposure has been linked to comorbidities such as hypertension, heart disease, and diabetes,

which can exacerbate respiratory decline (8). Evidence suggests that women exposed to biomass smoke may experience declines in lung function similar to those seen in smokers, characterized by

reduced forced expiratory volume in 1 second (FEV1), forced vital capacity (FVC), and an elevated risk of

developing COPD, and there is urgent need for tar- geted public health interventions aimed at reducing biomass smoke exposure among women in rural areas (9-11).

Individuals over 65 years of age exposed to biomass smoke experience an accelerated decline in respira- tory function, contributing to a more rapid progression of respiratory dysfunction (12). The Global Burden of Disease study emphasizes that elderly women, par- ticularly in rural settings, are at the highest risk for biomass smoke-related respiratory diseases (13). While the detrimental effects of biomass smoke on respiratory health have been well documented, few studies have specifically examined its impact on elderly women. This study aims to assess the pulmo- nary function of elderly women exposed to biomass smoke in rural areas, investigating the prevalence of obstructive lung conditions, as well as examine

demographic and comorbid factors associated with impaired lung function in this vulnerable population.

### MATERIALS and METHODS

**Participants**

In the village of Kağizman, a borough of Kars in east- ern Türkiye, a longstanding tradition designates women as responsible for baking bread within their households. This activity is often carried out under poor ventilation and in the absence of chimneys, resulting in significant exposure to smoke. Organic waste materials, particularly dried animal dung (manure), are commonly used as fuel sources instead of wood or charcoal. The primary inclusion criterion for the study was the screening of pulmonary func- tions in females with direct exposure to biomass smoke. Field visits to the village was conducted and evaluated a total of 474 females who met the expo- sure criteria. To ensure the accuracy of pulmonary

function test (PFT) results and minimize confounding factors, individuals with certain conditions or comor- bidities were excluded. Those were with a history of smoking, cardiovascular diseases, renal failure, asth- ma or allergic conditions and participants who had received treatment for upper or lower respiratory infections within the past month. Moreover, women with chronic pulmonary conditions, such as intersti- tial lung disease or bronchiectasis, significant tho- racic deformities, such as kyphoscoliosis; neuromus- cular disorders were also excluded (Figure 1). After applying these criteria, the final study cohort con- sisted of 115 females who were exclusively exposed to biomass smoke, of whom 32 women aged 65 years or older were identified and included in the study protocol (Figure 1). All the research procedures were approved by the local ethics committee of the Public Health Directorship in Kars, and all partici- pants provided written consent for the study.

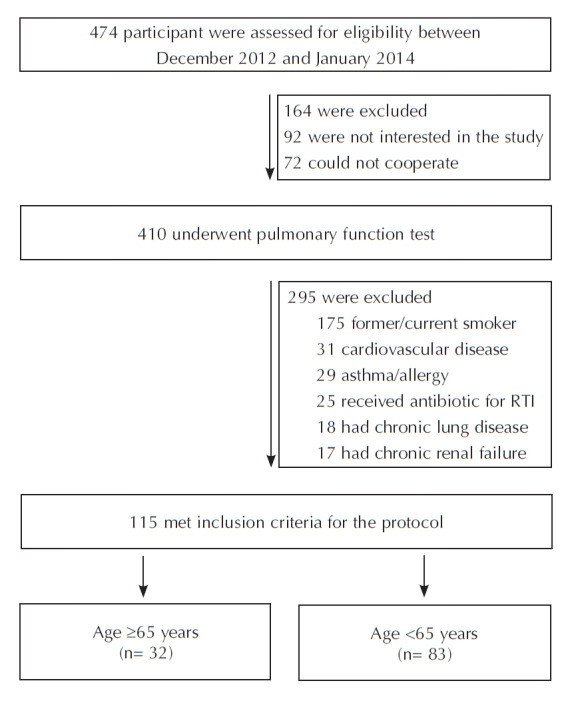

**Figure 1.** Flow chart of the participants.

### Pulmonary function testing

PFTs were performed using a calibrated Spirolab III device (MIR, Waukesha, WI, USA) under the super- vision of a trained technician. Measurements includ- ed FEV1, FVC, and FEV1/FVC ratio. These parameters were recorded for both the study and control groups. PFT results were categorized following the guide- lines of the European Respiratory Society and the American Thoracic Society and value of FEV1/FVC ratio below 70% was categorized as obstructive lung disease.

### Statistics

Continuous variables, such as age and BMI, were reported as mean ± standard deviation, while cate- gorical variables were expressed as frequencies and percentages. Comparisons between the groups were performed using independent-sample t-tests for nor- mally distributed continuous variables and the Mann- Whitney U test for non-normally distributed varia- bles. Chi-square or Fisher’s exact tests were used for categorical variables. Univariate logistic regression was used to evaluate the association between bio- mass smoke exposure duration and the presence of COPD. Variables with p< 0.05 in univariate regres- sion analysis were included in the multivariate regression model, which was adjusted for confound- ing factors. Odds ratios (ORs), β co-efficient with 95% confidence intervals (CIs) were reported to quantify the strength of associations. A p< 0.05 was considered statistically significant. All statistical anal- yses were performed using SPSS 28 (version 28.0 IBM Corp., Armonk, NY, USA).

## RESULTS

As described in Table 1, we compared pulmonary function, comorbidities, and exposure characteristics between women aged 65 years or older and those younger than 65 years. Mean age in the older group was significantly higher (71.0 ± 6.1 years) compared to the younger group (33.3 ± 12.5 years; p< 0.001). The starting age of biomass exposure did not differ significantly between the two groups (17.92 ± 3.21 years vs. 18.87 ± 3.43 years; p= 0.214). Hypertension was markedly more prevalent in the older group (84.6%) compared to the younger group (15.4%; p< 0.001), as was diabetes mellitus (34.6% vs. 6.7%; p<

0.001). An obstructive pulmonary pattern (FEV1/ FVC< 70%) was significantly more common among

women aged 65 years or older (76.9%) than in those younger than 65 years (15.7%; p< 0.001). The dura- tion of biomass smoke exposure was also signifi- cantly longer in the older group (41.1 ± 11.8 years) compared to the younger group (14.1 ± 11.9 years; p< 0.001). PFT results revealed that the older group had significantly lower FEV1/FVC ratios (0.67 ± 0.12

vs. 0.83 ± 0.09; p< 0.001), FEV1 percentages (40.23

± 9.72% vs. 66.79 ± 11.99%; p< 0.001), and FVC

percentages (59.15 ± 8.25% vs. 59.15 ± 8.25%; p< 0.001) compared to the younger group (Figure 2).

In Table 2, a multivariate logistic regression analysis was conducted to evaluate factors associated with obstructive pulmonary patterns among all partici- pants. Variables that were significant in the univariate analysis were included in the multivariate model, and we observed that the duration of biomass smoke exposure was significantly associated with obstruc- tion (OR= 1.25, 95% CI= 1.08-1.44, p< 0.001).

**Table:** 

**Table 1.** Comparison of demographic characteristics, comorbidities, and pulmonary function parameters between women aged ≥65 years and <65 years			
	**Age ≥65 years (n= 32)**	**Age <65 years (n= 83)**	**p**
Age, years	71.0 ± 6.1	33.3 ± 12.5	<0.001
Starting age	17.92 ± 3.21	18.87 ± 3.43	0.214
Hypertension, %	84.6	15.4	<0.001
Diabetes mellitus, %	34.6	6.7	<0.001
Obstructive pattern, %	76.9	15.7	<0.001
Duration, years	41.1 ± 11.8	14.1 ± 11.9	<0.001
FEV1/FVC	0.67 ± 0.12	0.83 ± 0.09	<0.001
FEV1, %	40.23 ± 9.72	66.79 ± 11.99	<0.001
FVC, %	59.15 ± 8.25	59.15 ± 8.25	<0.001
FEV1: Forced expiratory volume in 1 second, FVC: Forced vital capacity.

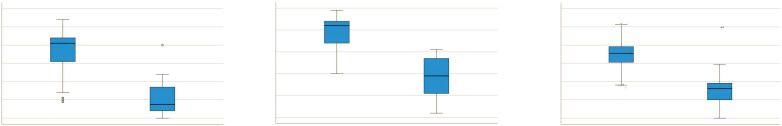
** Figure 2.** Comparison of pulmonary function test parameters (FEV1, FVC, and FEV1/FVC) between participants aged <65 years and

≥65 years.

**Table:** 

**Table 2.** Multivariate logistic regression analysis of factors associated with obstruction among all participants			
	**OR**	**95% CI**	**p**
Age	1.18	0.97-1.43	0.083
Age ≥65 years	0.04	0.01-2.07	0.108
Hypertension	0.47	0.04-6.18	0.565
Diabetes mellitus	1.62	0.15-18.11	0.695
**Duration**	**1.25**	**1.08-1.44**	**<0.001**
OR: Odds ratio, CI: Confidence interval.			

**Table:** 

**Table 3.** Linear regression FEV1/FVC ratio among all participants				
	β	**co-efficient**	**95% CI**	**p**
Age	-0.14		-0.03-0.01	0.380
Elderly	0.08		-0.04-0.08	0.417
Hypertension	0.06		-0.04-0.07	0.546
Diabetes mellitus	-0.05		-0.06-0.02	0.412
**Duration**	**-0.78**		**-0.08 to -0.04**	**<0.001**
CI: Confidence interval.

Other factors, including age, being aged ≥65 years, hypertension, and diabetes mellitus, were not statisti- cally significant in this model.

As illustrated in Table 3, a multivariate linear regres- sion analysis was performed to assess the relationship between FEV1/FVC ratio by adjusting the factors that were significant in the univariate model. Among the variables analyzed, the duration of biomass smoke exposure showed a significant negative association

with the FEV1/FVC ratio (β = -0.78, 95% CI= -0.08 to

-0.04, p< 0.001), indicating that longer exposure was

associated with a greater decline in this parameter. Other factors, including age, being elderly, hyperten- sion, and diabetes mellitus lost their significance in the multivariate analysis.

In Table 4 and Table 5, associates of obstruction and change in FEV1/FVC ratios were assessed. Among the variables analyzed, the duration of biomass smoke

**Table:** 

**Table 4.** Logistic regression analysis of factors associated with obstruction among elderly participants			
	**OR**	**95% CI**	**p**
Age	1.32	0.90-1.47	0.124
Hypertension	0.06	0.01-7.96	0.262
Diabetes mellitus	0.11	0.03-4.52	0.247
**Duration**	**1.26**	**1.02-1.57**	**0.035**
OR: Odds raito, CI: Confidence interval.			

**Table:** 

**Table 5.** Linear regression analysis of factors associated with FEV1/FVC ratio among elderly participants				
	β	**co-efficient**	**95% CI**	**p**
Age	-0.05		-0.07 to -0.05	0.748
Hypertension	0.25		-0.02-0.19	0.118
Diabetes mellitus	-0.05		-0.08-0.06	0.754
**Duration**	**-0.65**		**-0.09 to -0.03**	**<0.001**
CI: Confidence interval.

exposure was significantly associated with obstruc- tion (OR= 1.26, 95% CI= 1.02-1.57, p= 0.035) and

decline in the FEV1/FVC ratio (β= -0.65, 95% CI=

-0.09 to -0.03, p< 0.001). Other factors, including

age, hypertension and diabetes mellitus were not significantly associated with the obstruction as well as decline in FEV1/FVC ratio.

## DISCUSSION

The present study examines the impact of prolonged biomass smoke exposure on pulmonary function among women, with a particular focus on those aged 65 years and older. Our findings indicate that elderly women exhibit significantly lower pulmonary func- tion parameters, and a higher prevalence of obstruc- tive lung patterns compared to their younger counter- parts.

Mean age of biomass smoke exposure initiation did not differ significantly between the two groups, sug- gesting that the duration of exposure, rather than the age at which exposure began, plays a more critical role in pulmonary function decline. This is consistent with existing literature indicating that cumulative exposure to biomass smoke is a significant risk factor for developing chronic respiratory conditions, such as COPD (14). The higher prevalence of comorbidi- ties, such as hypertension and diabetes mellitus, among the older cohort aligns with general epide- miological trends observed in aging populations. However, the interplay between these comorbidities and biomass smoke exposure may exacerbate res- piratory decline, as systemic conditions like hyper- tension and diabetes can adversely affect lung func- tion and amplify the detrimental effects of environ-

mental pollutants (15). The significantly lower FEV1, FVC, and FEV1/FVC ratios observed in the elderly group underscore the severe impact of prolonged

biomass smoke exposure on lung function. These findings are in line with previous studies that have reported similar declines in pulmonary function among individuals exposed to biomass smoke, inde-

pendent of smoking status (16-18). The predomi- nance of obstructive lung patterns in the older group suggests that biomass smoke exposure may lead to airway obstruction, a hallmark of COPD. This is cor- roborated by studies indicating that biomass smoke exposure increases the risk of developing COPD, with a significant association observed between bio- mass smoke exposure and COPD in both rural and urban women (14).

The duration of biomass smoke exposure emerged as the only significant factor associated with an increased likelihood of obstruction. This finding rein- forces the established link between prolonged bio- mass smoke exposure and obstructive lung diseases, such as COPD, a relationship supported by prior studies demonstrating that biomass exposure inde- pendently contributes to the development of COPD in non-smoking populations (19-21). Other variables such as demographics and comorbidities were not significant predictors of COPD, these findings suggest that the adverse effects of biomass smoke exposure on pulmonary obstruction are not primarily mediated by those factors but are directly attributable to the cumulative exposure itself (3,22). The accumulation of harmful PM 2.5, CO, and other toxic compounds from biomass combustion induces chronic airway inflammation, oxidative stress, and structural dam- age, leading to progressive respiratory impairment (23).

Prolonged biomass smoke exposure on pulmonary health outcomes among elderly women has also been evaluated, and the duration of biomass smoke exposure has been reported as a significant predictor of obstructive pulmonary patterns among participants over 65 years. This finding aligns with the existing literature indicating that cumulative exposure to bio- mass smoke is a critical, moreover an independent risk factor for developing COPD (15). Other variables such as age, hypertension, and diabetes mellitus were not significant predictors in this model, and this

suggests that the adverse effects of biomass smoke exposure on pulmonary obstruction are primarily attributable to the cumulative exposure itself, rather than being mediated by demographic or comorbid factors.

This study has several limitations that warrant consid- eration. The cross-sectional design prevents estab- lishing a causal relationship between biomass smoke exposure and pulmonary function decline. Additionally, self-reported data on the duration and intensity of exposure may be subject to recall bias, potentially affecting the accuracy of the exposure assessment. The study was conducted in a single rural region, which may limit the generalizability of the findings to other populations with differing environ- mental and socio-economic conditions. Furthermore, the sample size of elderly participants, while ade- quate for statistical analysis, may not capture the full variability of pulmonary outcomes across broader populations. Last, while efforts were made to control for confounders such as age and comorbidities, unmeasured factors like dietary habits, genetic pre- dispositions, or co-exposures (e.g., occupational hazards) could influence the results. Future studies with longitudinal designs, larger sample sizes, and more detailed exposure assessments are recom- mended to build on these findings.

This study highlights the significant impact of pro- longed biomass smoke exposure on pulmonary func- tion, particularly among elderly women in rural set- tings. The findings demonstrate that cumulative expo- sure is strongly associated with both obstructive pul-

monary patterns and reduced FEV1/FVC ratios, inde- pendent of other demographic or comorbid factors.

These results emphasize the urgent need for targeted public health interventions, including the promotion of cleaner cooking technologies, improved ventila- tion, and educational programs to reduce biomass smoke exposure. By addressing this critical environ- mental health issue, policymakers can help mitigate the burden of chronic respiratory diseases and improve the quality of life for vulnerable populations.

**Ethical Committee Approval:** This study was approved by the Ethics Committee of the Kars Provincial Directorate of Health, Public Health Unit (Decision no: 7.2013.08, Date: 23.05.2013).

### CONFLICT of INTEREST

The authors declare that they have no conflict of interest.

## AUTHORSHIP CONTRIBUTIONS

Concept/Design: BT, BB Analys/Interpretation: BB, YC Data acqusition: YC

Writing: YK, SOY, BB Clinical Revision: SOY, BB Final Approval: All of authors

## REFERENCES

1. *Bruce N, Perez-Padilla R, Albalak R. Indoor air pollution in developing countries: A major environmental and public health challenge. Bull World Health Organ 2000; 78(9): 1078-92.*
2. *de Koning HW, Smith KR, Last JM. Biomass fuel combus- tion and health. Bull World Health Organ 1985; 63(1): 11-26.*
3. *Balcan B, Akan S, Ugurlu A, Handemir B, Ceyhan B, Ozkaya S. Effects of biomass smoke on pulmonary func- tions: A case control study. Int J Chron Obstruct Pulmon Dis 2016; 11: 1615-22. https://doi.org/10.2147/COPD.S109056*
4. *Gulsvik A. The global burden and impact of chronic obstructive pulmonary disease worldwide. Monaldi Arch Chest Dis 2001; 56(3): 261-4.*
5. *Naeher LP, Brauer M, Lipsett M, Zelikoff JT, Simpson CD, Koenig JQ, et al. Woodsmoke health effects: A review. Inhal Toxicol 2007; 19(1): 67-106. https://doi.org/10.1080/08958370600985875*
6. *Salvi SS, Barnes PJ. Chronic obstructive pulmonary disease in non-smokers. Lancet 2009; 374(9691): 733-43. https://doi.org/10.1016/S0140-6736(09)61303-9*
7. *Ortiz-Quintero B, Martínez-Espinosa I, Pérez-Padilla R. Mechanisms of Lung damage and development of COPD due to household biomass-smoke exposure: Inflammation, oxidative stress, microRNAs, and gene polymorphisms. Cells 2022; 12(1): 67. https://doi.org/10.3390/cells12010067*
8. *Burroughs Peña M, Romero KM, Velazquez EJ, Davila- Roman VG, Gilman RH, Wise RA, et al. Relationship between daily exposure to biomass fuel smoke and blood pressure in high-altitude Peru. Hypertension 2015; 65(5): 1134-40. https://doi.org/10.1161/HYPERTENSIONAHA.114.04840*
9. *Adeloye D, Song P, Zhu Y, Campbell H, Sheikh A, Rudan I. Global, regional, and national prevalence of, and risk fac- tors for, chronic obstructive pulmonary disease (COPD) in 2019: A systematic review and modelling analysis. Lancet Respir Med 2022; 10(5): 447-58. https://doi.org/10.1016/S2213-2600(21)00511-7*
10. *Prüss-Ustün A, Bonjour S, Corvalán C. The impact of the environment on health by country: A meta-synthesis. Environ Health 2008; 7: 7. https://doi.org/10.1186/1476-069X-7-7*
11. *Gut-Gobert C, Cavaillès A, Dixmier A, Guillot S, Jouneau S, Leroyer C, et al. Women and COPD: Do we need more evidence? Eur Respir Rev 2019; 28(151): 180055. https://doi.org/10.1183/16000617.0055-2018*
12. *Balmes JR. Household air pollution from domestic com- bustion of solid fuels and health. J Allergy Clin Immunol 2019; 143(6): 1979-87. https://doi.org/10.1016/j.jaci.2019.04.016*
13. *Balakrishnan K, Sankar S, Parikh J, Padmavathi R, Srividya K, Venugopal V, et al. Daily average exposures to respira- ble particulate matter from combustion of biomass fuels in rural households of southern India. Environ Health Perspect 2002; 110(11): 1069-75. https://doi.org/10.1289/ehp.021101069*
14. *Adama S, Serge MAS, Nicolas M, Catherine B. Chronic obstructive pulmonary disease associated with biomass fuel use in women: A systematic review and meta-analysis. BMJ Open Respiratory Research 2018; 5(1): e000246. https://doi.org/10.1136/bmjresp-2017-000246*
15. *Salahuddin M, Khan DA, Ayub S, Shahzad T, Irfan M. Biomass smoke-associated lung diseases. Current Pulmonology Reports 2023; 12(3): 151-61. https://doi.org/10.1007/s13665-023-00318-9*
16. *Viramgami A, Sheth A, Bagepally BS, Balachandar R. Study on the association between domestic biomass fuel exposure and pulmonary function: A systematic review and meta-analysis. Air Qual Atmos Health 2023; 16(12): 2529-51. https://doi.org/10.1007/s11869-023-01421-1*
17. *Hu G, Zhou Y, Tian J, Yao W, Li J, Li B, et al. Risk of COPD from exposure to biomass smoke: A metaanalysis. CHEST 2010; 138(1): 20-31. https://doi.org/10.1378/chest.08-2114*
18. *Sana A, Somda SMA, Meda N, Bouland C. Chronic obstructive pulmonary disease associated with biomass fuel use in women: A systematic review and meta-analysis. BMJ Open Respir Res 2018; 5(1): e000246. https://doi.org/10.1136/bmjresp-2017-000246*
19. *Salvi S, Barnes PJ. Is exposure to biomass smoke the big- gest risk factor for COPD globally? CHEST 2010; 138(1): 3-6. https://doi.org/10.1378/chest.10-0645*
20. *Ibtissam ELH, Vanessa GL, Soumaya B, Chakib N, Mohammed ElB, Mohamed Chakib B, et al. Association between biomass exposure and COPD occurrence in Fez, Morocco: Results from the BOLD study. BMJ Open Respir Res 2024; 11(1): e002409. https://doi.org/10.1136/bmjresp-2024-002409*
21. *El-Essawy AF, Ali MA, Al-Sherbiny NA, Elhefny RA, Farhat ES. Chronic obstructive pulmonary disease among women using biomass fuels in some rural areas of Fayoum gover- norate. Egypt J Bronchol 2015; 9(3): 227-30. https://doi.org/10.4103/1687-8426.165891*
22. *Capistrano SJ, Van Reyk D, Chen H, Oliver BG. Evidence of biomass smoke exposure as a causative factor for the development of COPD. Toxics 2017; 5(4): 36. https://doi.org/10.3390/toxics5040036*
23. *Ortiz-Quintero B, Martínez-Espinosa I, Pérez-Padilla R. Mechanisms of lung damage and development of COPD due to household biomass-smoke exposure: Inflammation, oxidative stress, microRNAs, and Gene polymorphisms. Cells 2023; 12(1): 67. https://doi.org/10.3390/cells12010067*

